# HEALTH RISKS AMONG OLDER ADULTS ACCORDING TO PRIMARY LANGUAGE: EVIDENCE FROM MEDICARE ANNUAL WELLNESS VISITS

**DOI:** 10.1093/geroni/igac059.1185

**Published:** 2022-12-20

**Authors:** Amjed Alsulaily, Kristen Berg, Lorella Luezas-Shamakian, Douglas Einstadter, Adam Perzynski

**Affiliations:** Case Western Reserve University, Qatif, Ash Sharqiyah, Saudi Arabia; The MetroHealth System, Cleveland, Ohio, United States; The MetroHealth System, Cleveland, Ohio, United States; The MetroHealth System, Cleveland, Ohio, United States; MetroHealth and Case Western Reserve University, Cleveland, Ohio, United States

## Abstract

Emerging evidence suggests that older adults benefit from the Medicare Annual Wellness Visit (AWV). Lind and colleagues found that between 2011 and 2016, utilization of the AWV increased from 8.1% to 23.0%. However, compared with non-Hispanic whites, AWV utilization is 11.6% points lower for Hispanic/Latinos and 10.2% lower for non-Hispanic Blacks. AWV differences by primary language have not been previously reported. We examined the rate of AWV utilization for older adults with English vs. Limited English Proficiency (LEP) among patients seen at a large urban health system in northeastern Ohio. Using Bonferroni-corrected chi-square and t-tests, we also evaluated the association between LEP and health risks (e.g., depression, falls, activities of daily living, cognitive status) assessed during the AWV. Using the electronic health record, we identified 41,262 Medicare patients aged 65+ who were eligible for the AWV. Of those identified, 42.8% completed an AWV between 2019 to 2021. Persons who were white (41.7%), Hispanic (37%), male (39.9%), Spanish-speaking (37.2%) or other LEP (41.2%) had lower utilization of AWVs (p<.001). Pain and fatigue ratings, depressive symptoms, and oral health problems and were similar between language groups, while cognitive impairment (p<.001), functional independence (p<.001), and self-rated health (p<.001) were substantially worse among non-English speakers. LEP is associated with a lower rate of AWV utilization and worse self-rated health. A clearer understanding of how speaking a different language from that in which the AWV is conducted is important for clarifying discrepancies and disparities in minority population health.

